# Supplementary material for: Nighttime lights as a proxy for conflict intensity and infrastructure recovery in Yemen and Ukraine
Source: Confl Health. 2026 May 11;20:59. doi: 10.1186/s13031-026-00801-5 (PMC13335313; doi:10.1186/s13031-026-00801-5)
Supplement: Supplementary file 1 — Additional file 1. [file 13031_2026_801_MOESM1_ESM.pdf]

Supplementary Material

Map of Yemen (Fig S1) 2
Map of Ukraine (Fig S2) 3
Comparison of view angles (Fig S3) 4
Data quality and completeness (Fig S4) 5
Interpolation sensitivity analysis (Fig S5, Tab S1) 6
Secondary population analysis (Fig S6, Tab S2) 7
Population sensitivity analysis (Fig S7–8, Tab S3–5) 9
BFAST simulations (Fig S9–13, Tab S6) 13
Residual plots (Fig S14–15) 19
Equations 21
Raymah sensitivity analysis (Tab S7) 23
Cholera model with NTL ratio (Fig S16–17, Tab S8) 24
Pre- and post-event means (Fig S18) 27
BFAST breakpoints (Tab S9–10) 28
Splines for NTL and attack models (Fig S19) 30
Splines for cholera model (Fig S20) 31
Stabilization blackout sensitivity analysis (Fig S21) 32

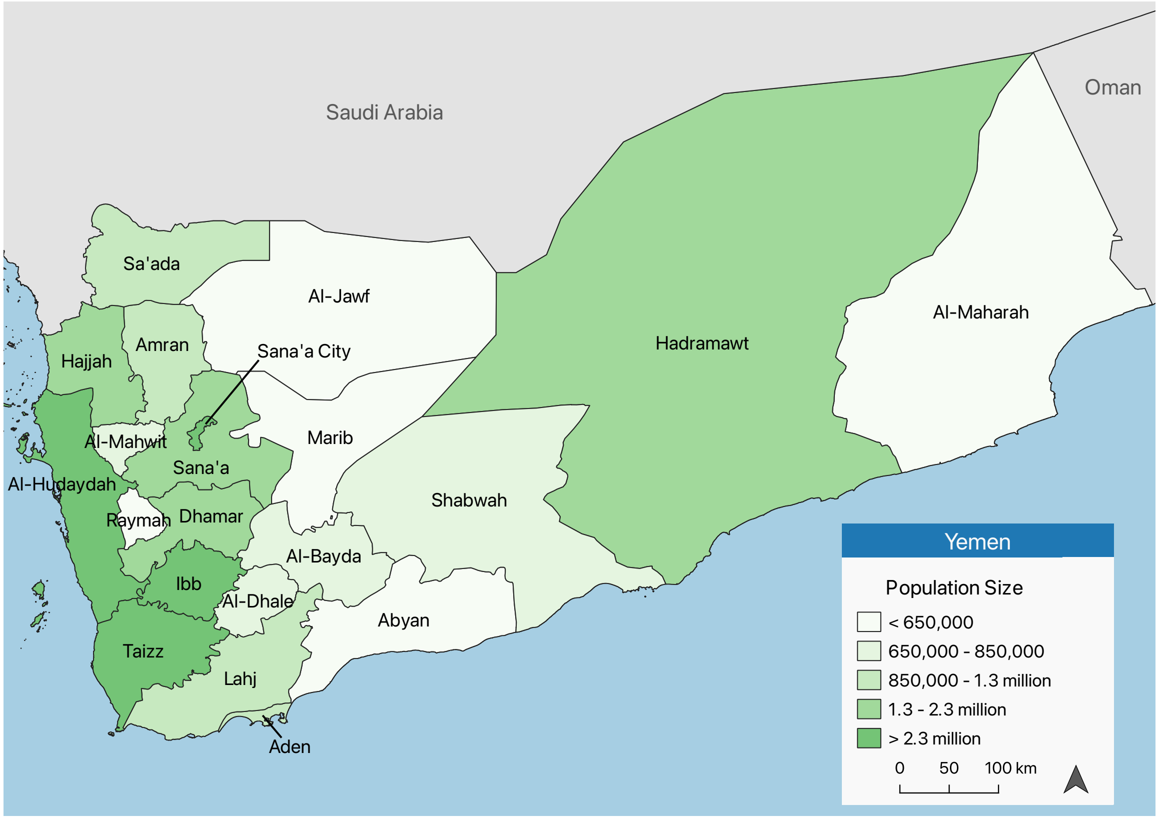

Fig. S1. Map of Yemen with mean estimated population size over the study period. Map made in QGIS v.3.34

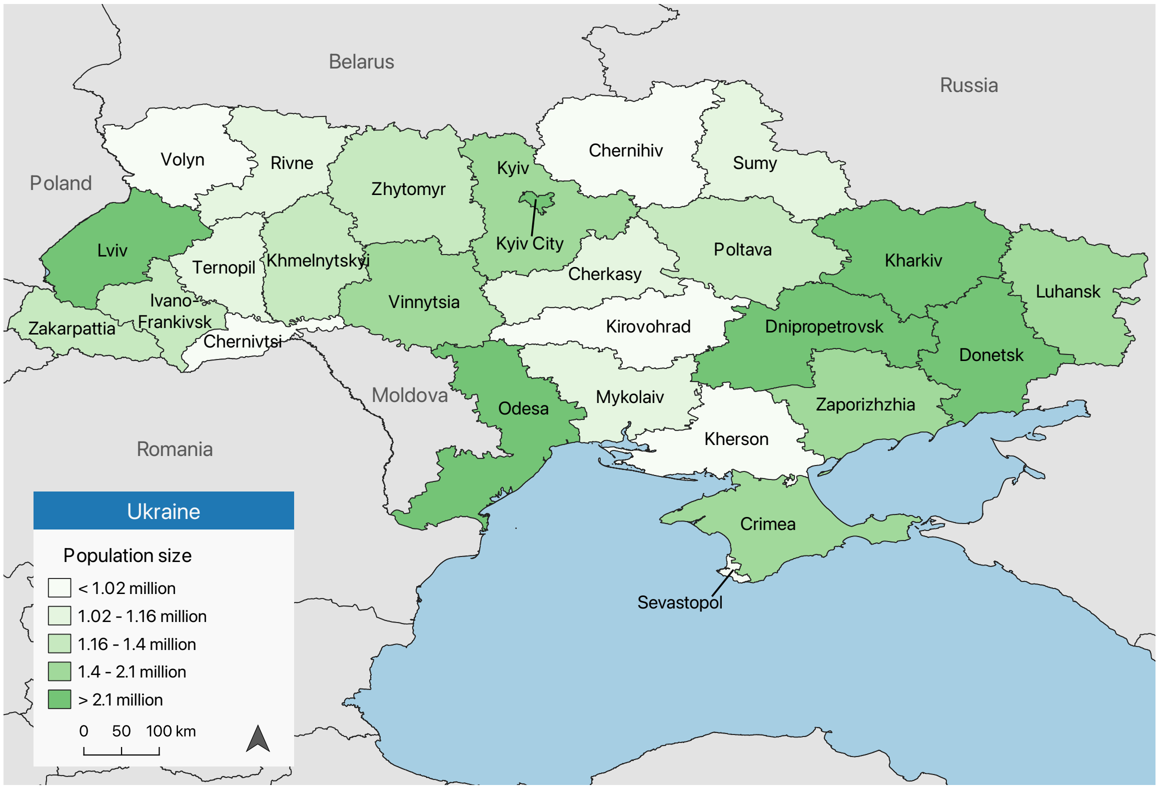

Fig. S2. Map of Ukraine with mean estimated population size over the study period. Map made in QGIS v.3.34

Comparison of view angles

Black Marble provides monthly NTL data generated from three view zenith angle (VZA) categories: near-nadir, off-nadir, and all-angles. Near-nadir composites utilize high-quality observations at VZA ≤ 20° (directly above), and off-angle composites utilize observations at VZA ≥ 40° (at an angle). All-angles composites are derived from all angular observations (i.e., a combination of near-nadir and off-nadir data) (1,2). Prior work indicates that each angle detects different luminosity levels (which may in part reflect different sources of light emission) and have varying stability (1,2). For this study, we chose to use the all-angles dataset as it is a middle ground in both radiance and stability compared to other view angles. This is especially relevant as we were not attempting to detect radiance from specific light sources and had a large overall spatial scale. All Black Marble data were extracted using *blackmarbleR* in R v.4.4.2 (3).

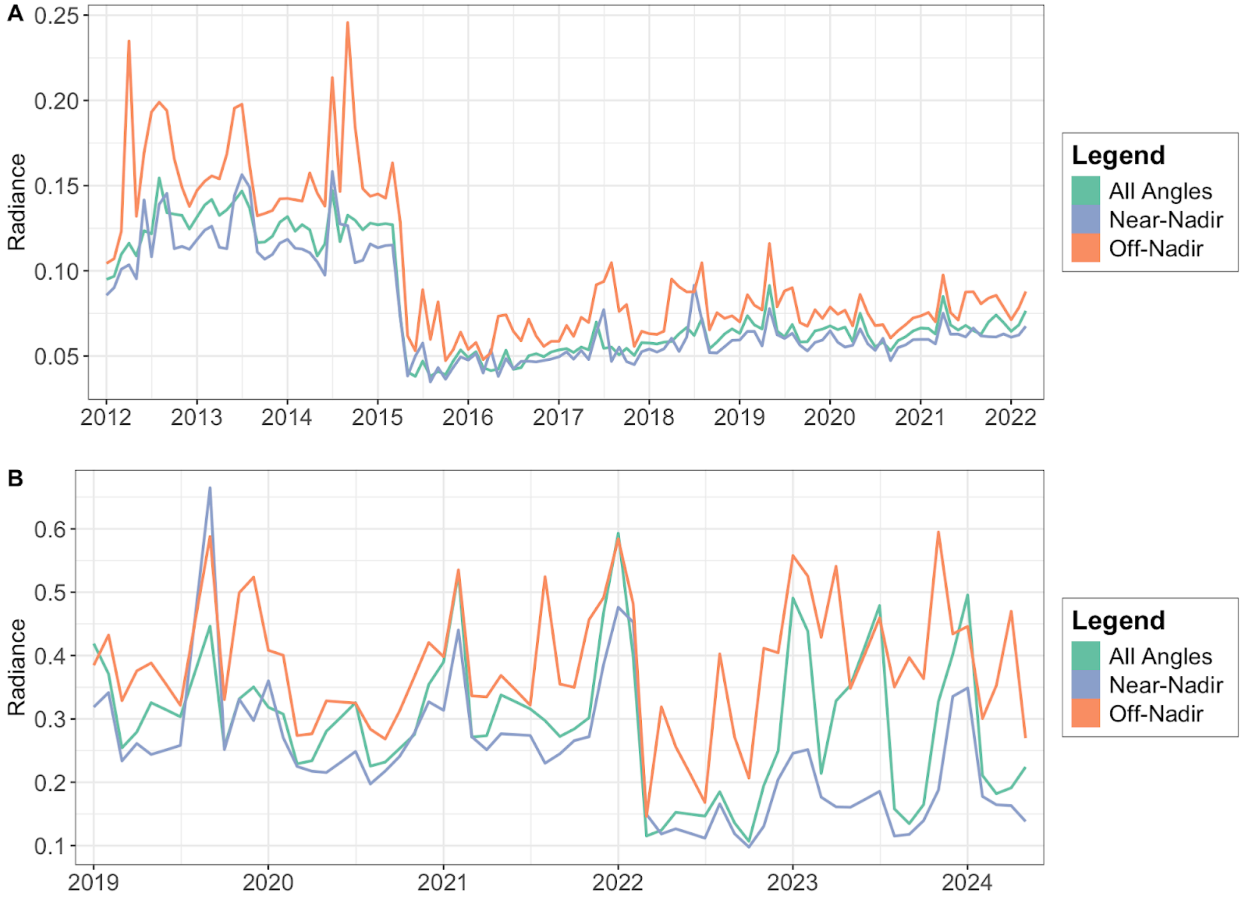

Fig. S3. Plot of available view zenith angles for NASA Black Marble in Yemen (A) and Ukraine (B).

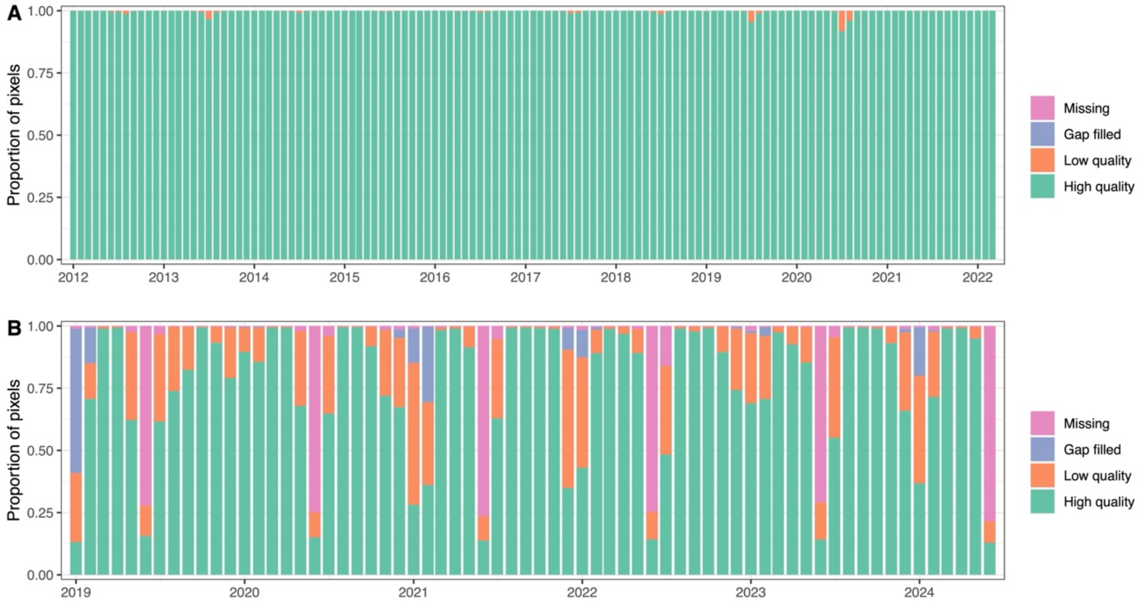

Fig. S4. Data quality and completeness of monthly NTL data for Yemen (A) and Ukraine (B). Black Marble defines high-quality pixels as those informed by more than 3 observations, and low-quality pixels as those with 3 or fewer observations. Gap-filled pixels are based on historical data. In Ukraine, the large variability in quality and missingness likely stems from seasonality (i.e., fewer snow-free observations in winter months to inform the composite) and Black Marble's stray light correction protocols in northern latitudes. Given this reported missingness in the summer months, we interpolated those NTL values for analysis.

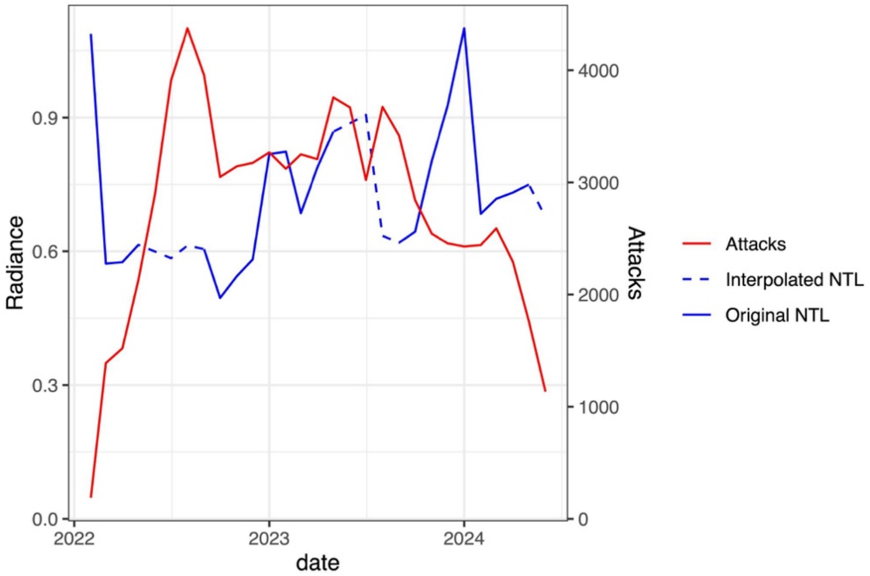

Fig. S5. Comparison of original mean NTL time series, interpolated data, and total number of aerial attacks in Ukraine.

	Mean NTL		NTL Ratio	
**Covariate**	**β (95% CI)**	**% change in outcome (95% CI)**	**β (95% CI)**	**% change in outcome (95% CI)**
**Ukraine**				
Aerial attacks				
None	Comparison		Comparison	
1 – 2	0.693 (0.581, 0.827)	-30.69% (-41.93%, -17.27%)	0.758 (0.646, 0.890)	-24.20% (-35.41%, -11.04%)
3 – 6	0.737 (0.598, 0.907)	-26.34% (-40.17%, -9.30%)	0.798 (0.661, 0.963)	-20.18% (-33.87%, -3.66%)
7 - 117	0.541 (0.414, 0.708)	-45.86% (-58.61%, -29.18%)	0.628 (0.495, 0.797)	-37.18% (-50.48%, -20.32%)
118 +	0.290 (0.203, 0.414)	-71.01% (-79.68%, -58.65%)	0.401 (0.294, 0.547)	-59.92% (-70.61%, -45.34%)

Table S1. Outputs of sensitivity analysis without NTL interpolation. NTL values in June–August of each year were left as missing (NA). These outputs do not meaningfully differ from those with interpolation.

Secondary population analysis

As a secondary analysis, we ran GAMs to measure the association between mean NTL and population size (as the response variable) in each administrative unit; these also included light recovery. This was primarily done to assess whether, in the absence of reliable population data, NTL could provide estimates of relative population size. The following GAMs were run using the *mgcv* package in R:

Yemen

$$pop = f_1(month) + f_2(perc\_built) + f_3(NTL_{mean}) + f_4(recovery) + \beta_1(attacks_{low}) + \beta_2(attacks_{medium}) + \beta_3(attacks_{high}) + \beta_4(attacks_{severe}) + f_5(lat, lon) \quad (1)$$

Where percentage built (*perc_built*), mean NTL (*NTL_{mean}),* and light recovery (*recovery*) were log-transformed and f_{1-5} are smooth functions estimated by the model using restricted maximum likelihood. Study month (*month*) was modeled using a cyclic cubic regression spline, centroid latitude and longitude (*lat,lon)* using two-dimensional splines on a sphere, and all others with thin plate regression splines. Aerial attack categories were defined as none (0 attacks), low (1–4), medium (5–12), high (13–35), and severe (36+). None was used as the reference category. Light recovery was originally tried as an interaction term with time since light nadir, but the interaction term was not meaningful and therefore not included.

Ukraine

$$pop = f_1(month) + f_2(NTL_{mean}) + f_3(recovery) + \beta_1(attacks_{low}) + \beta_2(attacks_{medium}) + \beta_3(attacks_{high}) + \beta_4(attacks_{severe}) + f_4(lat, lon) \quad (2)$$

Where mean NTL (NTL_{mean}) and light recovery (*recovery*) were log-transformed and f_{1-4} are smooth functions estimated by the model using restricted maximum likelihood. Study month (*month*) was modeled using a cyclic cubic regression spline, centroid latitude and longitude (*lat,lon)* using two-dimensional splines on a sphere, and all others with thin plate regression splines. Aerial attack categories were defined as none (0 attacks), low (1–2), medium (3–6), high (7–117), and severe (118+). None was used as the reference category. Light recovery was originally tried as an interaction term with time since light nadir, but the interaction term was not meaningful and therefore not included.

We found that NTL was positively associated with governorate and oblast population size. Conversely, recovery had a near-linear negative association with population size in both countries (**Figure S6**).

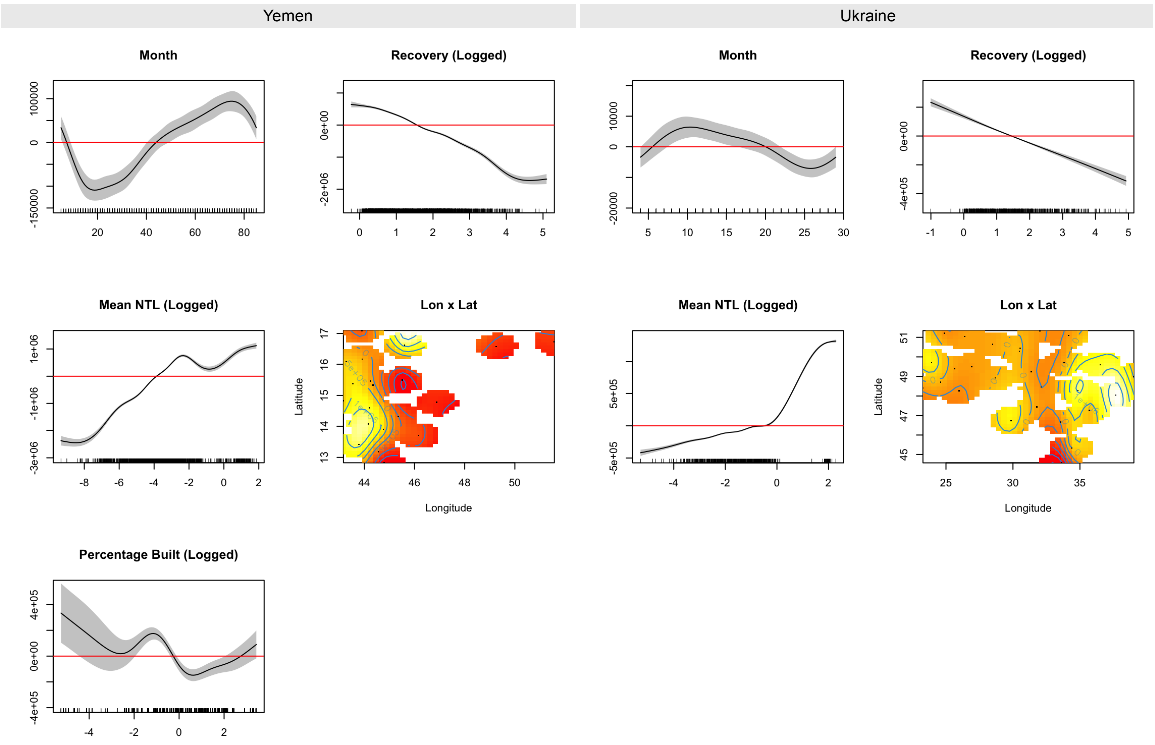

Fig. S6. Spline outputs for the NTL and population size GAMs for Yemen and Ukraine. The output is considered significant when the black line and gray shaded areas (95% CIs) are entirely above or below the red horizontal line. In interaction plots, yellows indicate higher population size and reds indicate lower population size.

In both countries, administrative units with more attacks had higher population levels, indicating that highly populated areas were more likely to be attacked (**Table S2**). Together with the spline outputs, this reflects that areas with higher population were unable to recover as strongly likely because they experienced more attacks.

	Yemen	Ukraine
**Covariate**	***β* (95% CI)**	***β* (95% CI)**
Aerial attacks		
Low	27,933.00 (-722.20–56,688.20)	4,743.00 (-635.20–10,121.20)
Medium	60,374.00 (26,983.40–93,764.60)	1,248.00 (-5,555.20–8,051.20)
High	70,243.00 (31,666.30–108,819.70)	27,263.00 (16,140.00–38,386.00)
Severe	-9,722.00 (-52,485.30–33,041.30)	28,294.00 (12,604.20–43,983.80)

Table S2. Linear outputs for the NTL and population size GAMs for Yemen and Ukraine.

Sensitivity analysis of population size estimates

To address any concerns over potential endogeneity in our models, we ran sensitivity analyses using population estimates from Gridded Population of the World (GPW) in place of WorldPop estimates. WorldPop population estimates are informed by NTL data, though the exact scope and scale of NTL data use in the population estimates used in our analyses is unclear. GPW provides non-modeled population estimates that are based on historical census data and do not use NTL data; estimates are available every five years between 2000 and 2015. We ran the sensitivity analyses using the unadjusted and UN-adjusted estimates. To create annual measurements, we used two techniques: 1) applying the growth rate between 2015 and 2020 to all years between 2015 and 2022, and 2) applying the mean growth rate of the entire 2000–2015 period to all years between 2015 and 2022. This resulted in four population estimates for sensitivity analyses. Results for Yemen's NTL and aerial attacks model can be found in **Tables S3** and **S4**, and outputs for the secondary population estimate and NTL models are in **Table S5** and **Figures S7** and **S8**. There are no meaningful differences from the models that use WorldPop data, indicating that endogeneity is not a concern in our models. We have therefore opted to use WorldPop data in the main text because it is available annually and thus requires less interpolation.

	**Gridded Population of the World (unadj.)**		**Gridded Population of the World (adj.)**	
**Covariate**	**Interp. 1 (2015–2020 growth rate)**	**Interp. 2 (Average growth rate)**	**Interp. 1 (2015–2020 growth rate)**	**Interp. 2 (Average growth rate)**
		**β (95% CI)**		
Air raids				
Individual	0.997 (0.995, 0.999)	0.997 (0.995, 0.999)	0.997 (0.995, 0.999)	0.997 (0.995, 0.999)
Scaled	0.919 (0.874, 0.967)	0.919 (0.874, 0.967)	0.916 (0.872, 0.964)	0.918 (0.873, 0.965)
Diesel (10% change)	0.975 (0.963, 0.987)	0.975 (0.963, 0.987)	0.976 (0.964, 0.989)	0.975 (0.963, 0.987)
		**% change in outcome (95% CI)**		
Air raids				
Individual	-0.31% (-0.49%, -0.12%)	-0.31% (-0.49%, -0.12%)	-0.32% (-0.51%, -0.14%)	-0.32% (-0.50%, -0.13%)
Scaled	-8.05% (-12.56%, -3.31%)	-8.05% (-12.56%, -3.31%)	-8.35% (-12.84%, -3.63%)	-8.22% (-12.71%, -3.49%)
Diesel (10% change)	-2.51% (-2.52%, -2.49%)	-2.51% (-2.52%, -2.49%)	-2.38% (-2.39%, -2.36%)	-2.49% (-2.51%, -2.47%)

Table S3. Outputs of sensitivity analysis showing the association between attacks and mean NTL. These results can be compared to those in **Table 1**, which use WorldPop population estimates.

Covariate	Gridded Population of the World (unadj.) Interp. 1 (2015–2020 growth rate)	Gridded Population of the World (unadj.) Interp. 2 (Average growth rate)	Gridded Population of the World (adj.) Interp. 1 (2015–2020 growth rate)	Gridded Population of the World (adj.) Interp. 2 (Average growth rate)
***β* (95% CI)**				
Air raids				
Individual	0.996 (0.993, 0.998)	0.996 (0.993, 0.998)	0.995 (0.993, 0.998)	0.995 (0.993, 0.998)
Scaled	0.885 (0.829, 0.945)	0.885 (0.829, 0.945)	0.883 (0.827, 0.943)	0.884 (0.828, 0.944)
Diesel (10% change)	0.976 (0.960, 0.993)	0.976 (0.960, 0.993)	0.976 (0.960, 0.992)	0.976 (0.960, 0.993)
**% change in outcome (95% CI)**				
Air raids				
Individual	-0.45% (-0.69%, -0.21%)	-0.45% (-0.69%, -0.21%)	-0.46% (-0.70%, -0.22%)	-0.45% (-0.69%, -0.21%)
Scaled	-11.49% (-17.10%, -5.49%)	-11.49% (-17.10%, -5.49%)	-11.72% (-17.32%, -5.73%)	-11.59% (-17.20%, -5.60%)
Diesel (10% change)	-2.37% (-2.39%, -2.34%)	-2.37% (-2.39%, -2.34%)	-2.40% (-2.42%, -2.38%)	-2.37% (-2.40%, -2.35%)

Table S4. Outputs of sensitivity analysis showing the association between attacks and NTL ratio. These results can be compared to those in **Table 1**, which use WorldPop population estimates.

Covariate	Gridded Population of the World (unadj.) Interp. 1 (2015–2020 growth rate)	Gridded Population of the World (unadj.) Interp. 2 (Average growth rate)	Gridded Population of the World (adj.) Interp. 1 (2015–2020 growth rate)	Gridded Population of the World (adj.) Interp. 2 (Average growth rate)
Yemen				
Aerial attacks				
Low	20,978.00 (-8,429.84, 50,385.84)	20,978.00 (-8,429.84, 50,385.84)	21,451.00 (-7,641.28, 50,543.28)	21,348.00 (-8,295.04, 50,991.04)
Medium	55,972.00 (21,752.36, 90,191.64)	55,971.00 (21,751.36, 90,190.64)	58,309.00 (24,444.12, 92,173.88)	57,234.00 (22,738.00, 91,730.00)
High	61,923.00 (22,350.60, 101,495.40)	61,924.00 (22,351.60, 101,496.40)	66,378.00 (27,234.84, 105,521.20)	63,839.00 (23,951.04, 103,727.00)
Severe	-27,136.00 (-71,057.64, 16,785.64)	-27,135.00 (-71,056.64, 16,786.64)	-18,652.00 (-62,089.52, 24,785.52)	-25,093.00 (-69,361.56, 19,175.56)
Ukraine				
Aerial attacks				
Low	4,015.00 (-451.84, 8,481.84)	4,009.00 (-520.56, 8,538.56)	3,998.00 (-464.92, 8,460.92)	3,986.00 (-535.72, 8,507.72)
Medium	1,096.00 (-4,554.68, 6,746.68)	1,136.00 (-4,593.08, 6,865.08)	1,129.00 (-4,517.76, 6,775.76)	1,151.00 (-4,568.28, 6,870.28)
High	22,250.00 (13,008.60, 31,491.40)	22,298.00 (12,929.20, 31,666.80)	22,270.00 (13,036.44, 31,503.56)	22,261.00 (12,907.88, 31,614.12)
Severe	23,930.00 (10,894.04, 36,965.96)	23,895.00 (10,676.76, 37,113.24)	23,977.00 (10,950.84, 37,003.16)	23,908.00 (10,713.28, 37,102.72)

Table S5. **Outputs of sensitivity analysis for the secondary NTL and population size models**. These results can be compared to those in **Table S2**, and spline outputs are shown in **Figures S7 and S8**.

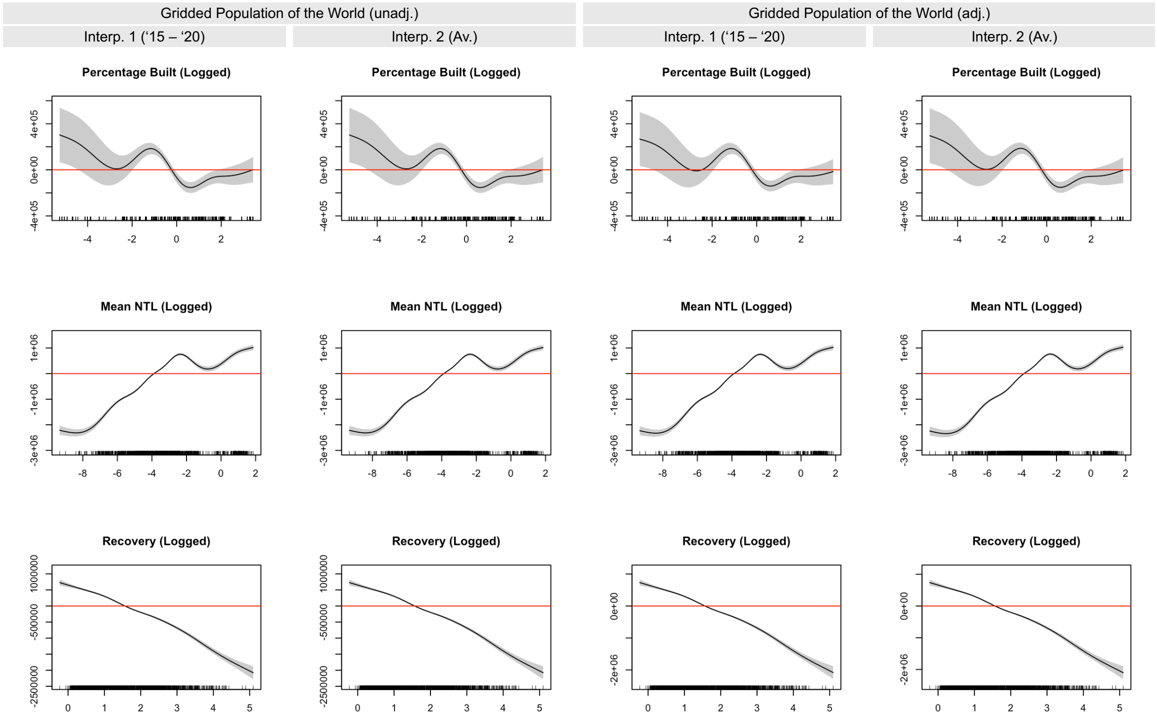

Fig. S7. Select spline outputs for the NTL and population size sensitivity analysis for Yemen. The model also controls for month and location, though these are not shown for brevity; they are not meaningfully different from those shown in **Figure S6**. The output is considered significant when the black line and gray shaded areas (95% CIs) are entirely above or below the red horizontal line. The parametric outputs for these models are shown in **Table S5** and can be compared to the outputs from the model using WorldPop population estimates in **Figure S6 and Table S2.**

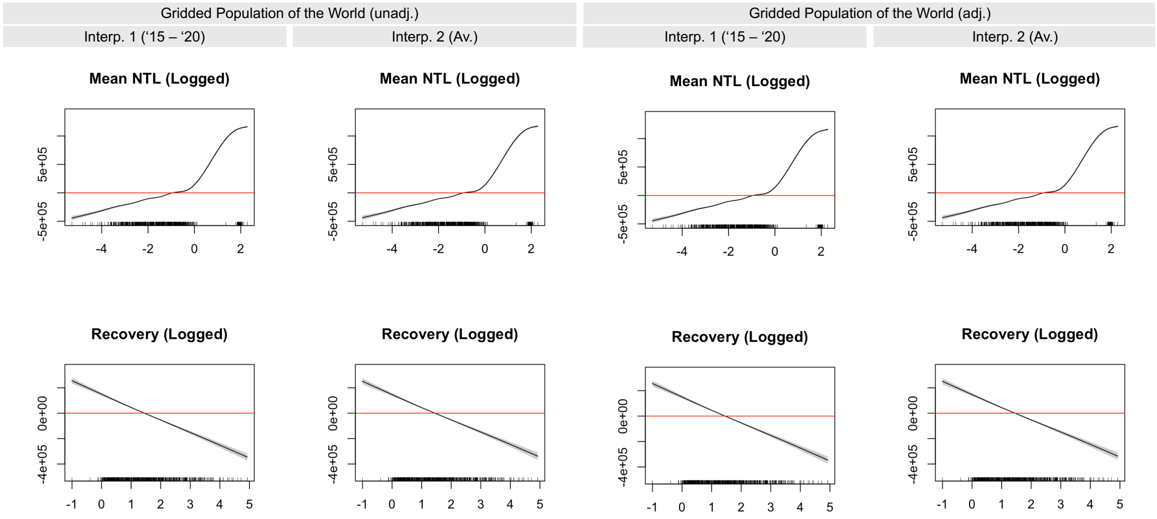

Fig. S8. Select spline outputs for the NTL and population size sensitivity analysis for Ukraine. The model also controls for month and location, though these are not shown for brevity; they are not meaningfully different from those shown in **Figure S6**. The output is considered significant when the black line and gray shaded areas (95% CIs) are entirely above or below the red horizontal line. The parametric outputs for these models are shown in **Table S5** and can be compared to the outputs from the model using WorldPop population estimates in **Figure S6 and Table S2.**

BFAST simulations

We used the Breaks for Additive Season and Trend (BFAST) change detection approach to identify negative breakpoints in the mean NTL time series for each administrative unit to assess whether the onset of attacks was evident in units' NTL signals. BFAST decomposes time series into seasonal, trend, and noise components while estimating the number, timing, magnitude, and direction of significant changes within them. To validate our use of BFAST, we simulated monthly NTL time series that were informed by the real data (see **Methods** for more detail). Parameters used in the simulations are in **Table S6,** and examples of the simulated time series are in **Figure S9.** Simulations were first run over all trend values while holding noise values for each period constant (**Figures S10** and **S11**). We then ran a second set of simulations that held the trend values for each period constant and ran over all noise values (**Figure S12** and **S13**). Importantly, the pre, peri, and post values for both trend and noise are quite similar in all the simulations, meaning there was often minimal signal in the data to detect change.

	**Yemen-informed simulations**	**Ukraine-informed simulations**
Trend		
Pre-break	0.07, 0.11, 0.15, 0.19, 0.23, 0.27	0.15, 0.20, 0.25, 0.30, 0.40, 0.45, 0.50, 0.60
Peri-break	0.005, 0.01, 0.02, 0.03, 0.04	0.10, 0.12, 0.15, 0.17, 0.20, 0.25, 0.30
Post-break	0.01, 0.03, 0.05, 0.07, 0.09	0.15, 0.20, 0.25, 0.30, 0.40, 0.45
Noise		
Pre-break	0.01, 0.02, 0.03, 0.04, 0.05	0.04, 0.05, 0.06, 0.07, 0.08
Peri-break	0.005, 0.008, 0.011, 0.014	0.04, 0.05, 0.06, 0.07, 0.08, 0.09
Post-break	0.005, 0.01, 0.015, 0.02	0.06, 0.07, 0.08, 0.09, 0.10, 0.11
Pulse	[0, 0, 0.002, 0.004, -0.004, 0.003, 0, -0.004]	[0.017, -0.032, 0.008, 0.047, 0.025, -0.026, -0.003, -0.012]
Seasonality	[-0,001, -0.0002, 0.0011, 0.0003, -0.0025, 0, 0.0038, 0.0006, -0.0025, -0.002, 0.0021, 0.002]	[0.1414, 0.0653, -0.0439, -0.056, -0.0042, 0.0168, -0.0121, -0.0282, -0.0602, -0.0693, -0.0256, 0.0994]
Breakpoints	18, 19, 20, 21…105	9, 10, 11, 12…57

Table S6. Parameters used in BFAST simulations. Parameters were informed by decomposing each administrative unit's real NTL time series; the trend and noise parameters encompass the median and quartile values across all administrative units in each country. The pulse and seasonality parameters are the median values across all administrative units. Note that in the simulations, individual values were selected for the trend, noise, and breakpoint, and the pulse and seasonality values were included as a block. Ten simulations were generated for each set of parameters at every breakpoint, resulting in 164,640 simulations informed by the Ukraine data and 132,000 by the Yemen data.

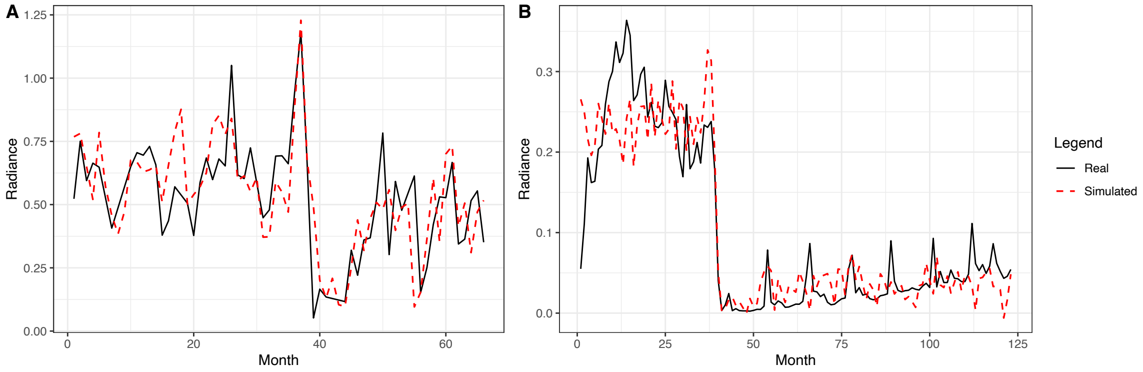

Fig. S9. Examples of simulated time series compared to real data from Ukraine (A) and Yemen (B).

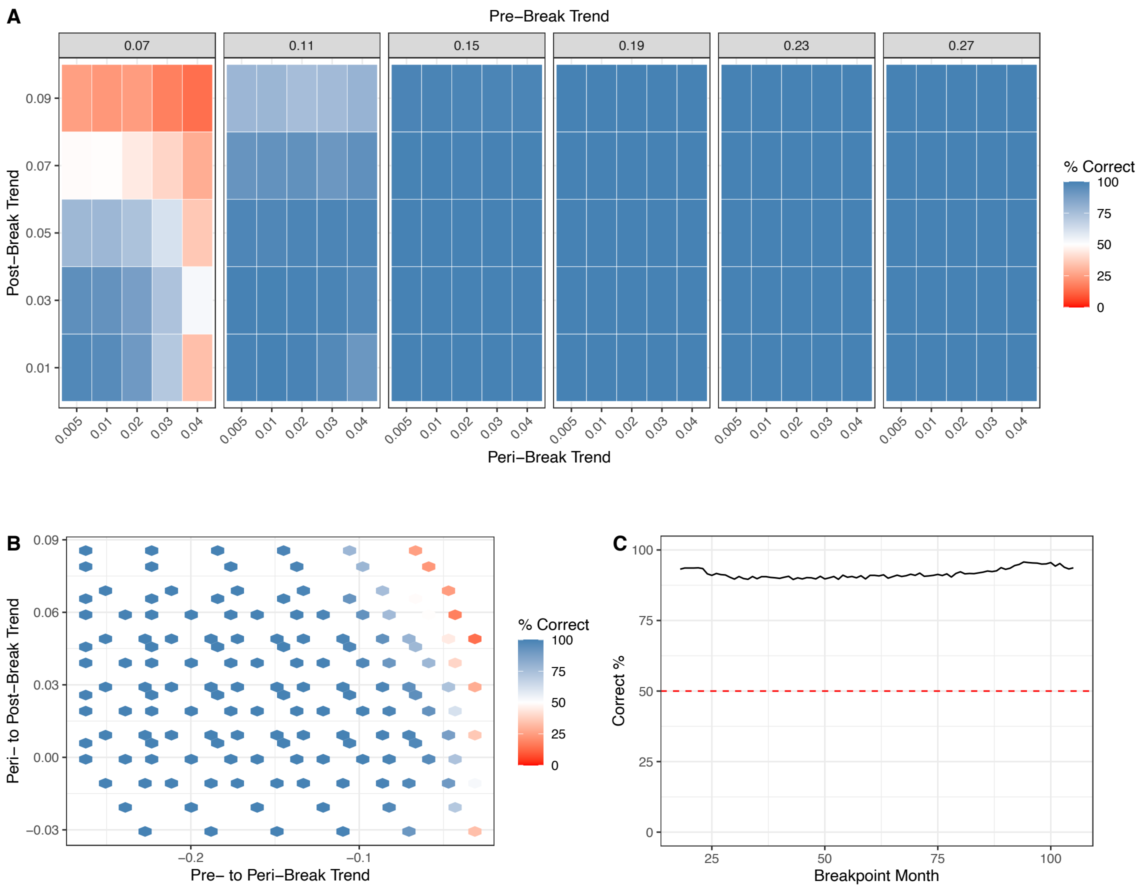

Fig. S10. Yemen-informed simulations with changing trend values. A) Heat plot showing percentage of simulations (n=132,000) in which BFAST correctly detected the breakpoint by pre-break NTL trend values (i.e., each box is labeled by pre-break trend level). Across all grids, x-axes show peri-break trend values and y-axes show post-break trend values. Noise values for each period were held at the median parameter values (0.027, 0.008, and 0.011 respectively). B) Plot showing percentage of correct BFAST detections by difference in trend between periods. Values on the x-axis are the difference in trend values from the pre-break to the peri-break periods, whereby negative values indicate a decrease in the trend from the pre- to the peri-break period. Values on the y-axis are the difference in trend values between the peri-break and post-break periods, with positive values indicating an increase in the trend from the peri- to the post-break period. C) Percentage of correct BFAST detections by month of breakpoint over all simulations. The red horizonal dashed line shows a correct detection rate of 50%.

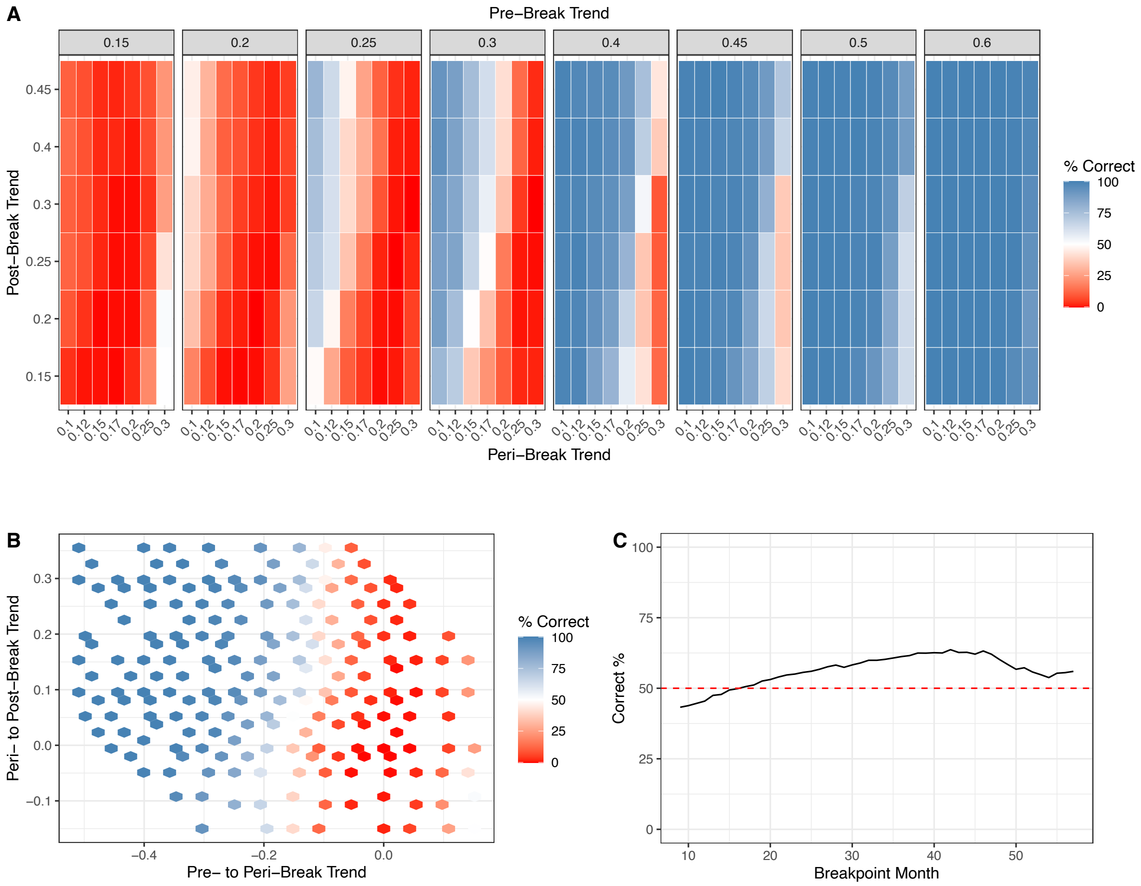

Fig. S11. Ukraine-informed simulations with changing trend values. A) Heat plot showing percentage of simulations (n=164,640) in which BFAST correctly detected the breakpoint by pre-break NTL trend values (i.e., each box is labeled by pre-break trend level). Across all grids, x-axes show peri-break trend values and y-axes show post-break trend values. Noise values for each period were held at the median parameter values (0.060, 0.069, and 0.090 respectively). B) Plot showing percentage of correct BFAST detections by difference in trend between periods. Values on the x-axis are the difference in trend values between the pre-break and peri-break periods, whereby negative values indicate a decrease in the trend from the pre- to the peri-break period. Values on the y-axis are the difference in trend values between the peri-break and post-break periods, with positive values indicating an increase in the trend from the peri- to the post-break period. C) Percentage of correct BFAST detections by month of breakpoint over all simulations. The red horizonal dashed line shows a correct detection rate of 50%. We hypothesize that the non-linear shape indicates that BFAST performs better when breakpoints are surrounded by available data rather than in proximity to unavailable data (i.e., the end points); this may be especially relevant in these simulations given the shorter time series length compared to the Yemen-informed simulations (66 versus 123 months).

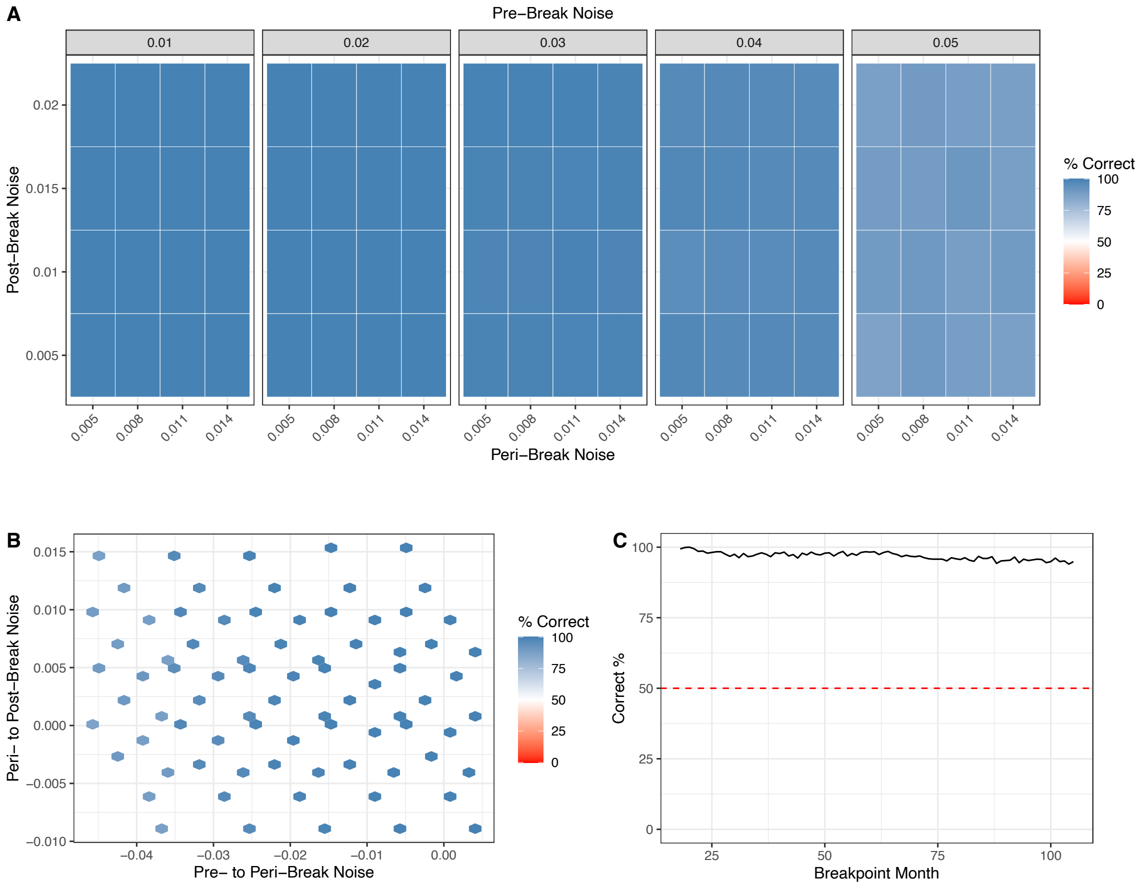

Fig. S12. Yemen-informed simulations with changing noise values. A) Heat plot showing percentage of simulations (n=70,400) in which BFAST correctly detected the breakpoint by pre-break noise values (i.e., each box is labeled by pre-break noise level). Across all grids, the x-axes show peri-break noise values and y-axes show post-break noise values. Trend values for each period were held at the median parameter values (0.110, 0.019, and 0.031 respectively). B) Plot showing percentage of correct BFAST detections by difference in noise between periods. Values on the x-axis are the difference in noise values between the pre-break and peri-break periods, whereby negative values indicate a decrease in noise from the pre- to the peri-break period. Values on the y-axis are the difference in noise values between the peri-break and post-break periods, with positive values indicating an increase in noise from the peri- to the post-break period. C) Percentage of correct BFAST detections by month of breakpoint over all simulations. The red horizonal dashed line shows a correct detection rate of 50%.

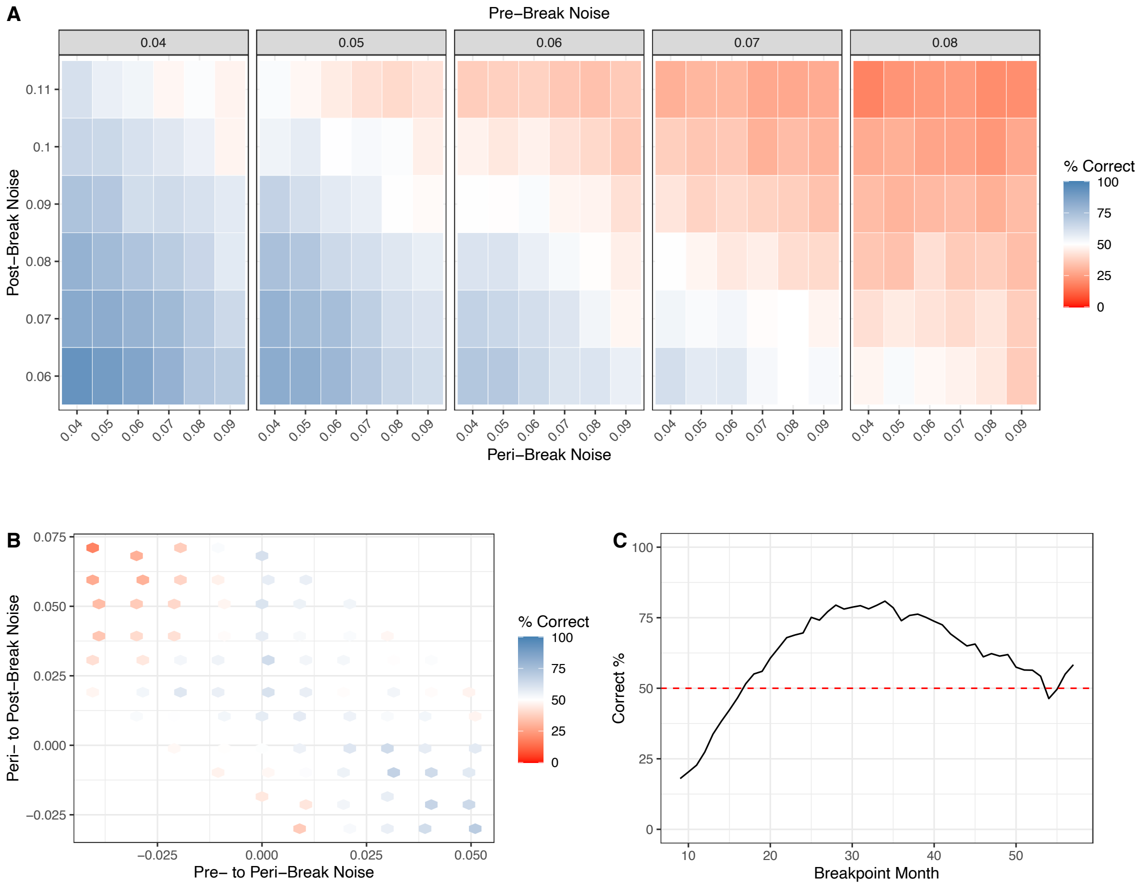

Fig. S13. Ukraine-informed simulations with changing noise values. A) Heat plot showing percentage of simulations (n=88,200) in which BFAST correctly detected the breakpoint by pre-break noise values (i.e., each box is labeled by pre-break noise level). Across all grids, the x-axes show peri-break noise values and y-axes show post-break noise values. Trend values for each period were held at the median parameter values (0.282, 0.165, and 0.241 respectively). B) Plot showing percentage of correct BFAST detections by difference in noise between periods. Values on the x-axis are the difference in noise values between the pre-break and peri-break periods, whereby negative values indicate a decrease in noise from the pre- to the peri-break period. Values on the y-axis are the difference in noise values between the peri-break and post-break periods, with positive values indicating an increase in noise from the peri- to the post-break period. C) Percentage of correct BFAST detections by month of breakpoint over all simulations. The red horizonal dashed line shows a correct detection rate of 50%. We hypothesize that the non-linear shape indicates that BFAST performs better when breakpoints are surrounded by available data rather than in proximity to unavailable data (i.e., the end points); this may be especially relevant in these simulations given the shorter time series length compared to the Yemen-informed simulations (66 versus 123 months).

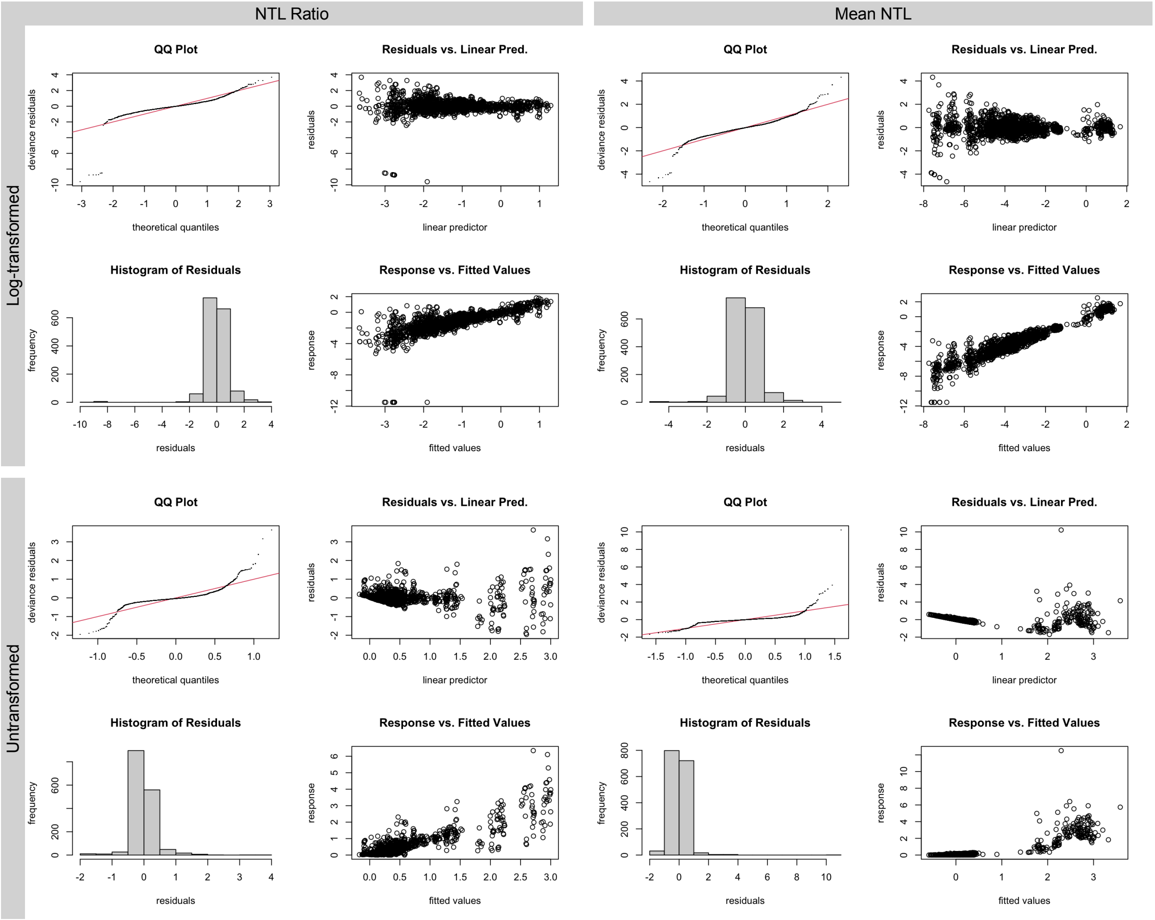

Fig. S14. Residual plots for the GAM used to measure the relationship between NTL and aerial attacks in Yemen. In the GAMs modeling the relationship between NTL and aerial attacks, mean NTL and NTL ratio (as the response variable) were both log-transformed to handle skewness and based on model residuals. The top row shows the residuals when NTL (both mean and ratio) as the response is log-transformed, and the bottom row shows the residuals when NTL is not transformed. The residuals for the models with NTL log-transformed indicate a better fit.

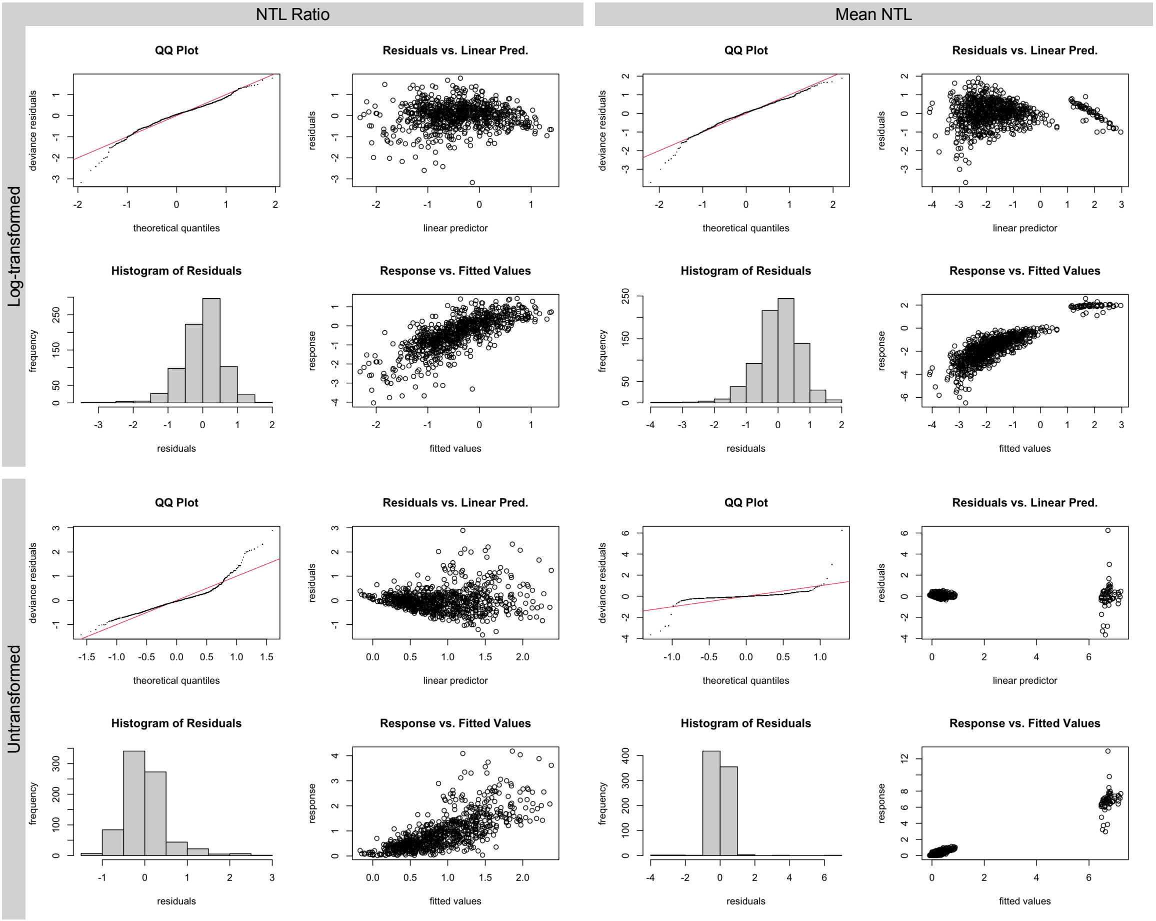

Fig. S15. Residual plots for the GAM used to measure the relationship between NTL and aerial attacks in Ukraine. In the GAMs modeling the relationship between NTL and aerial attacks, mean NTL and NTL ratio (as the response variable) were both log-transformed to handle skewness and based on model residuals. The top row shows the residuals when NTL (both mean and ratio) as the response is log-transformed, and the bottom row shows the residuals when NTL is not transformed. The residuals for the models with NTL log-transformed indicate a better fit.

Equations: Nighttime lights and aerial attacks

We ran the following generalized additive models (GAMs) with a Gaussian distribution to analyze the relationship between nighttime lights and aerial attacks. GAMs were run in the *mgcv* package in R v.4.4.2 (4). Linear outputs for each model can be found in **Table 1** and spline outputs in **Figure S19**.

Yemen

$$\log(NTL_{mean}) = \beta_1(attacks) + f_1(month) + f_2(pop) + f_3(perc\_built) + \beta_2(diesel) + f_4(lat, lon) \quad (1)$$

$$\log(NTL_{ratio}) = \beta_1(attacks) + f_1(month) + f_2(pop) + f_3(perc\_built) + \beta_2(diesel) + f_4(lat, lon) \quad (2)$$

Where population (*pop*), percentage built (*perc_built*), and diesel (*diesel*) were log-transformed and f_{1-4} are smooth functions estimated by the model using restricted maximum likelihood. Study month (*month*) was modeled using a cyclic cubic regression spline, *pop* and *perc_built* with thin plate regression splines, and centroid latitude and longitude (*lat,lon)* using two-dimensional splines on a sphere. Each model was run with the raw number of aerial attacks (*attacks*) and with the number of attacks mean-centered and standardized (mean: 14, SD: 27).

Ukraine

$$\log(NTL_{mean}) = \beta_1(attacks_{low}) + \beta_2(attacks_{medium}) + \beta_3(attacks_{high}) + \beta_4(attacks_{severe}) + f_1(month) + f_2(perc\_built) + f_3(lat, lon) \quad (3)$$

$$\log(NTL_{ratio}) = \beta_1(attacks_{low}) + \beta_2(attacks_{medium}) + \beta_3(attacks_{high}) + \beta_4(attacks_{severe}) + f_1(month) + f_2(perc\_built) + f_3(lat, lon) \quad (4)$$

Where f_{1-3} are smooth functions estimated by the model using restricted maximum likelihood. *Month* was modeled using a cyclic cubic regression spline, *perc_built* with a thin plate regression spline, and *lat,lon* using two-dimensional splines on a sphere. Aerial attack categories were defined as none (0 attacks), low (1–2), medium (3–6), high (7–117), and severe (118+). None was used as the reference category.

Equation: Cholera model

We applied nighttime lights-defined conflict categories to an existing model assessing the relationship between conflict and cholera in Yemen between 2016 and 2019. Details on the original work can be found here (5). GAMs were run in the *mgcv* package in R v.4.4.2 with a negative binomial distribution (4). Linear outputs are in **Table 2**, plots of the light recovery interactions (f_5) are in **Figure 3**, and other spline outputs are in **Figure S20**.

Without recovery

$$\begin{aligned} cases = {} & f_1(pop\_density) + f_2(week) + f_3(lat, lon) + \beta_1(conflict_{interm.}) \\ & + \beta_2(conflict_{medium}) + \beta_3\left(conflict_{high}\right) + \beta_4(conflict_{severe}) + \beta_5(precip) \\ & + \beta_6(surf\_wat) + \beta_7(temp) + \beta_8(NDVI) + f_4(economy) \end{aligned} \quad (5)$$

With recovery

$$\begin{aligned} cases = {} & f_1(pop\_density) + f_2(week) + f_3(lat, lon) + \beta_1(conflict_{interm.}) \\ & + \beta_2(conflict_{medium}) + \beta_3\left(conflict_{high}\right) + \beta_4(conflict_{severe}) + \beta_5(precip) \\ & + \beta_6(surf\_wat) + \beta_7(temp) + \beta_8(NDVI) + f_4(economy) \\ & + f_5(recovery, months\_since\_min) \end{aligned} \quad (6)$$

Conflict categories were defined using air raids in the original model and with mean NTL in the NTL-based model. Air raid-defined categories were defined as low (zero air raids in prior 3 months), intermediate (1–4), medium (5–18), high (19–75), and severe (≥76). Mean NTL-based categories were defined as low (0.087–5.904 $nW \cdot cm^{-2} \cdot sr^{-1}$), intermediate (0.031–0.086), medium (0.016–0.030), high (0.007–0.015), and severe (0–0.006). f_{1-5} are smooth functions estimated by the model using restricted maximum likelihood. Study week (*week*) was modeled using a cyclic cubic regression spline, centroid latitude and longitude (*lat,lon)* using two-dimensional splines on a sphere, and all others with thin plate regression splines.

	**Original Model**<br>*No. gov. = 20*	**Original Model**<br>*No. gov. = 19*<br>*(ex. Raymah)*	**Mean NTL Model**<br>**(with recovery)**<br>*No. gov. = 19*
**Covariate**	**IRR (95% CI)**	**IRR (95% CI)**	**IRR (95% CI)**
Conflict severity			
Low	Comparison	Comparison	Comparison
Intermediate	1.76 (1.51, 2.05)	1.90 (1.61, 2.25)	1.34 (1.06, 1.69)
Medium	1.57 (1.31, 1.89)	1.54 (1.26, 1.87)	1.89 (1.42, 2.52)
High	1.87 (1.53, 2.29)	2.09 (1.69, 2.59)	1.94 (1.38, 2.73)
Severe	2.06 (1.59, 2.69)	1.96 (1.49, 2.57)	1.71 (1.10, 2.64)
Surface water	0.89 (0.78, 1.03)	0.94 (0.81, 1.08)	0.90 (0.79, 1.02)
Precipitation	0.98 (0.91, 1.06)	1.03 (0.95, 1.11)	1.10 (1.02, 1.19)
Vegetation index	0.72 (0.64, 0.80)	0.75 (0.65, 0.85)	0.74 (0.66, 0.84)
Temperature	1.11 (0.91, 1.36)	1.01 (0.83, 1.23)	1.10 (0.92, 1.32)

Table S7. Model outputs for original cholera model and models without Raymah governorate. To assess whether the exclusion of Raymah from the NTL-based model biased the results, we ran the original model (using air raids to define conflict categories) without Raymah. These results were consistent with the full original model and the NTL-based model with light recovery, indicating minimal bias.

Cholera model with NTL ratio

We ran the cholera model, without recovery, using conflict categories defined by NTL ratios of low (0.54–0.99), intermediate (0.32–0.53), medium (0.17–0.31), high (0.10–0.16), and severe (0–0.09). We also created a separate category ("above") for NTL levels that were above pre-event means. We did not use this specification in a model with the recovery interaction term, as the NTL ratio already captures recovery over time relative to pre-event NTL levels. We included the model that combined mean NTL-defined conflict categories and the recovery interaction in the main text as it highlights the role of recovery in the epidemiological process more explicitly. However, the results from this model likewise indicate that areas with NTL levels closest to pre-event levels had the lowest cholera risk and that this risk increased as the ratio approached zero (**Table S8**).

We hypothesize that population displacement is captured in the "above" conflict category, which was associated with increased cholera risk (IRR: 1.51; 95% CI: 1.21–1.87) (**Table S8**). Most of the included governorate-weeks were from Marib, Al-Jawf, and Al-Maharah; as of 2023, Marib hosts roughly 60% of Yemen's IDPs, or around 1.6 million people (6). Though Al-Jawf and Al-Maharah host far fewer IDPs (26,000 and 18,000, respectively), their pre-conflict light levels were considerably lower than that of other governorates (6). The influx of IDPs to these governorates may have had a greater effect on their NTL levels as the IDP developments accounted for a larger proportion of the overall light. We can use high-resolution satellite imagery to identify development in areas with observed NTL growth (**Figure S17**). Other governorates which hosted more IDPs started with higher light levels, and thus the impact of displacement may not have affected their overall means as strongly, though increases in specific areas may be detectable at smaller spatial scales.

	**Original Model**<br>*No. gov. = 20*		**NTL Ratio Model**<br>*No. gov. = 20*	
**Covariate**	**IRR (95% CI)**	**p-value**	**IRR (95% CI)**	**p-value**
Conflict severity				
Low	Comparison		Comparison	
Intermediate	1.76 (1.51–2.05)	<0.0001	1.29 (1.11–1.49)	0.0008
Medium	1.57 (1.31–1.89)	<0.0001	1.38 (1.18–1.62)	<0.0001
High	1.87 (1.53–2.29)	<0.0001	1.57 (1.30–1.90)	<0.0001
Severe	2.06 (1.59–2.69)	<0.0001	1.47 (1.18–1.83)	0.0005
Above			1.51 (1.21–1.87)	0.0002
Surface water	0.89 (0.78–1.03)	0.11	0.90 (0.78–1.03)	0.13
Precipitation	0.98 (0.91–1.06)	0.69	1.08 (0.99–1.17)	0.07
Vegetation index	0.72 (0.64–0.80)	<0.0001	0.70 (0.62–0.79)	<0.0001
Temperature	1.11 (0.91–1.36)	0.29	1.03 (0.85–1.25)	0.75
AIC		30,240.97		30,337.26

Table S8. Outputs for the original cholera model and the model using NTL ratio to define conflict categories. Spline outputs for the NTL-based model are in **Figure S16**.

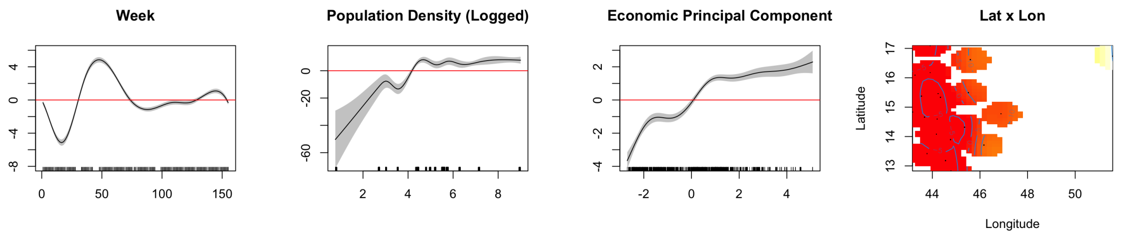

Fig. S16. Spline outputs of the cholera model using NTL ratios to define conflict categories.

“Above” Category

There were some governorates that had higher NTL values after the onset of large-scale military operations than before, which was captured in the “above” category in the NTL ratio. We hypothesize that this was capturing displacement, especially in areas that hosted a markedly high number of displaced individuals (e.g., Marib), or that had very low light levels before the onset of large-scale military operations, meaning that displaced settlements would have a proportionately larger effect on NTL levels. In **Figure S17**, we investigated two areas that saw higher NTL levels post-event: Al-Hazim in Al-Jawf governorate, and Marib City in Marib governorate. In Al-Hazim, increases in light levels reflect widespread development of the area, which previously had both low development and light levels. In Marib City, the creation of Jufainah camp, seen in the satellite image in **Figure S17**, likely contributed to the growth in NTL in the post-event period.

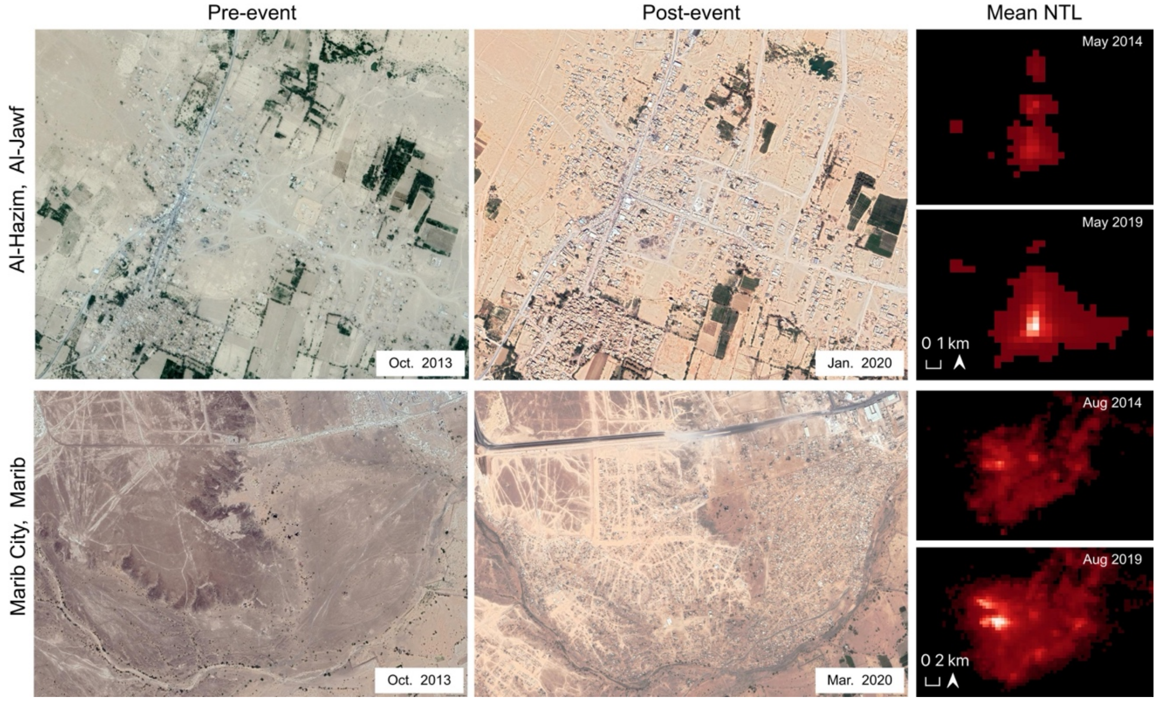

Fig. S17: Examples of displacement-related infrastructural development. The high-resolution satellite images were extracted from Google Earth.

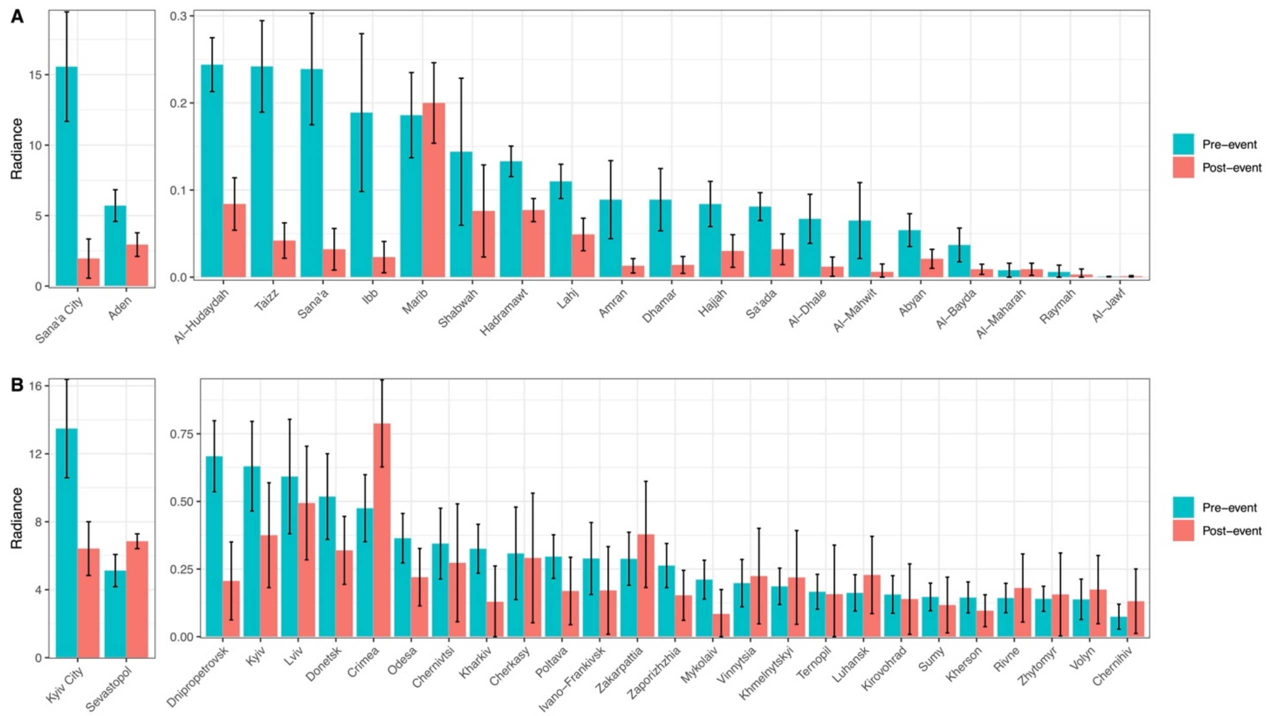

Fig. S18. Pre- and post-event NTL means and standard deviations in Yemen (A) and Ukraine (B)

	Month of first aerial attack	Breakpoint (95% CI)	h-value
Total Yemen	39 (Mar 2015)	39 (38–40)	0.15
Brightest quartile			
Sana'a City	39	39 (38–40)	0.15
Aden	39	39 (38–40)	0.15
Al-Hudaydah	39	39 (38–40)	0.15
Taizz	39	39 (38–40)	0.15
Sana'a	39	39 (38–40)	0.15
Second quartile			
Ibb	39	39 (38–40)	0.10
Marib	39	40 (38–41)	0.15
Shabwah	39	39 (38–40)	0.15
Hadramawt	43 (Jul 2015)	40 (38–41)	0.15
Lahj	39	39 (38–40)	0.15
Third quartile			
Amran	39	39 (38–40)	0.15
Dhamar	39	39 (37–40)	0.15
Hajjah	39	40 (38–41)	0.15
Sa'ada	39	40 (39–41)	0.15
Al-Dhale	39	39 (36–40)	0.15
Dimmest quartile			
Al-Mahwit	42 (Jun 2015)	40 (39–42)	0.10
Abyan	39	39 (38–40)	0.15
Al-Bayda	39	39 (38–40)	0.15
Al-Maharah	90 (Jun 2019)	None detected	–
Raymah	41 (May 2015)	None detected	–
Al-Jawf	39	39 (34–40)	0.10

Table S9: BFAST-identified negative breakpoints with 95% confidence intervals in Yemen. The h-value refers to the minimal segment size between potential breaks. Quartiles refer to pre-event NTL levels.

	Month of first aerial attack	Breakpoint (95% CI)	h-value
Ukraine	38 (Feb 2022)	38 (37–39)	0.15
Brightest Quartile			
Kyiv City	38	38 (37–39)	0.25
Sevastopol	43 (Jul 2022)	None detected	–
Dnipropetrovsk	38	38 (37–39)	0.10
Kyiv	38	38 (37–39)	0.15
Lviv	38	38 (37–39)	0.15
Donetsk	38	38 (37–40)	0.15
Second Quartile			
Crimea	44 (Aug 2022)	None detected	–
Odesa	38	None detected	–
Chernivtsi	46 (Oct 2022)	45 (44–46)	0.15
Kharkiv	38	38 (36–39)	0.20
Cherkasy	39 (Mar 2022)	37 (33–38)	0.15
Poltava	38	37 (36–41)	0.15
Ivano-Frankivsk	38	38 (36–39)	0.15
Third Quartile			
Zakarpattia	41 (May 2022)	47 (45–48)	0.10
Zaporizhzhia	38	38 (37–39)	0.15
Mykolaiv	38	None detected	–
Vinnytsia	38	None detected	–
Khmelnytskyi	39 (Mar 2022)	40 (38–41)	0.15
Ternopil	40 (Apr 2022)	40 (37–41)	0.15
Luhansk	38	None detected	–
Dimmest Quartile			
Kirovohrad	39	None detected	–
Sumy	38	38 (36–39)	0.05
Kherson	38	None detected	–
Rivne	39	None detected	–
Zhytomyr	38	38 (37–39)	0.05
Volyn	38	None detected	–
Chernihiv	38	44 (43–45)	0.10

Table S10: BFAST-identified negative breakpoints with 95% confidence intervals in Ukraine. The h-value refers to the minimal segment size between potential breaks. Quartiles refer to pre-event NTL levels.

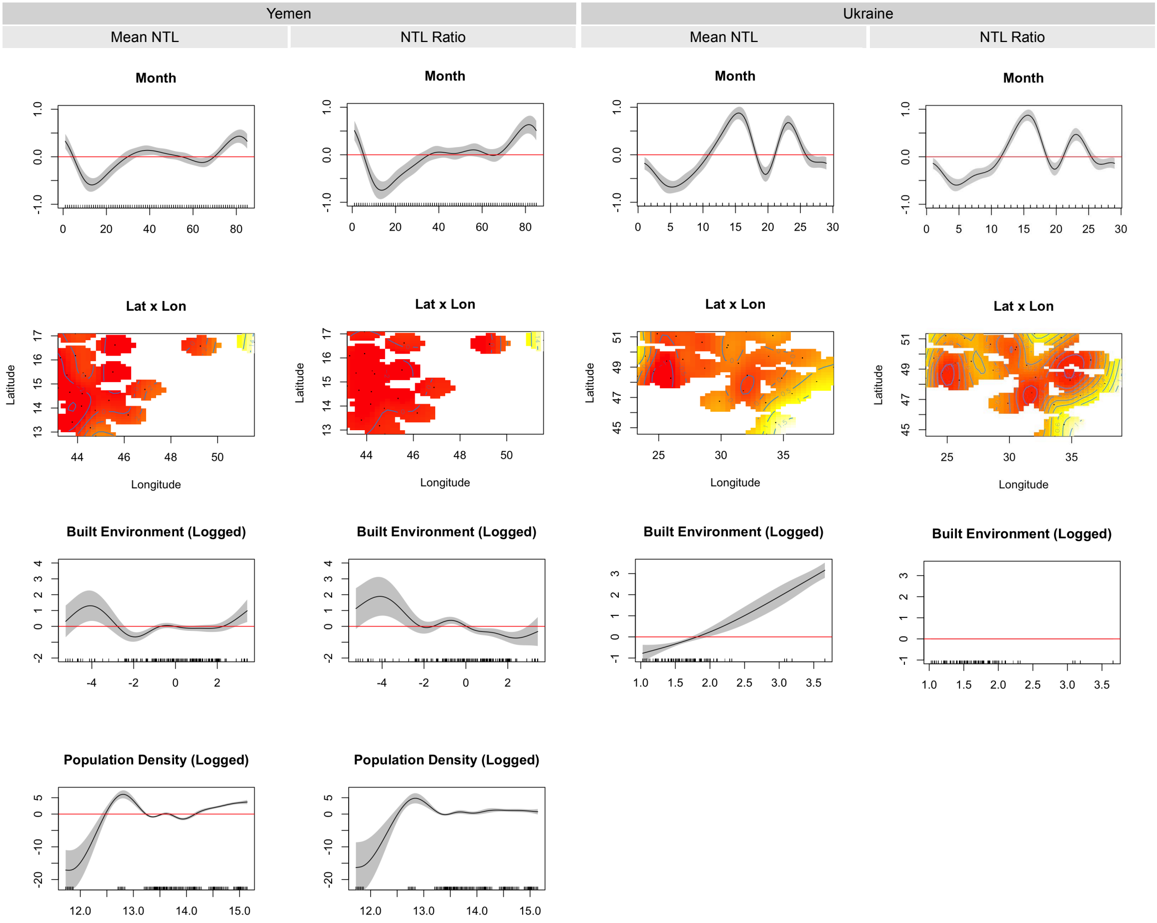

Fig. S19. Splines for the non-parametric variables included in the NTL and aerial attacks models. The output is considered significant when the black line and gray shaded areas (95% CIs) are entirely above or below the red horizontal line. In interaction plots, yellows indicate higher population size and reds indicate lower population size. The linear components for these models are in **Table 1.**

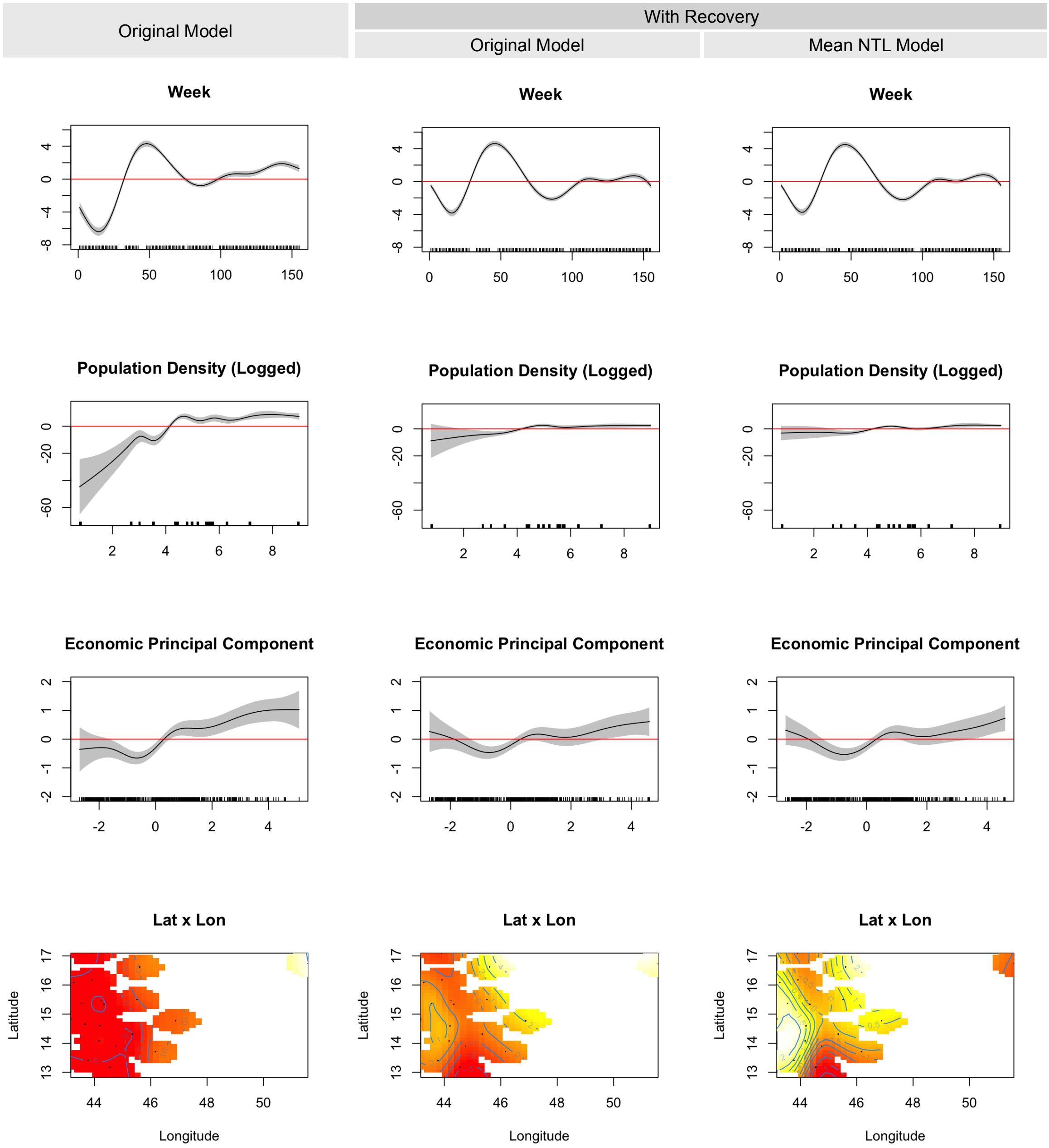

Fig. S20. Spline outputs for the cholera models. "Original" refers to the model from Tarnas et al. (5), which used air raids to define the conflict categories. Models with recovery included an interaction term between light recovery and months since light nadir. The output is considered significant when the black line and gray shaded areas (95% CIs) are entirely above or below the red horizontal line. In interaction plots, yellows indicate higher population size and reds indicate lower population size. The linear outputs are shown in **Table 2** and the interaction plots for light recovery (in the "With Recovery" models) are shown in **Figure 3**.

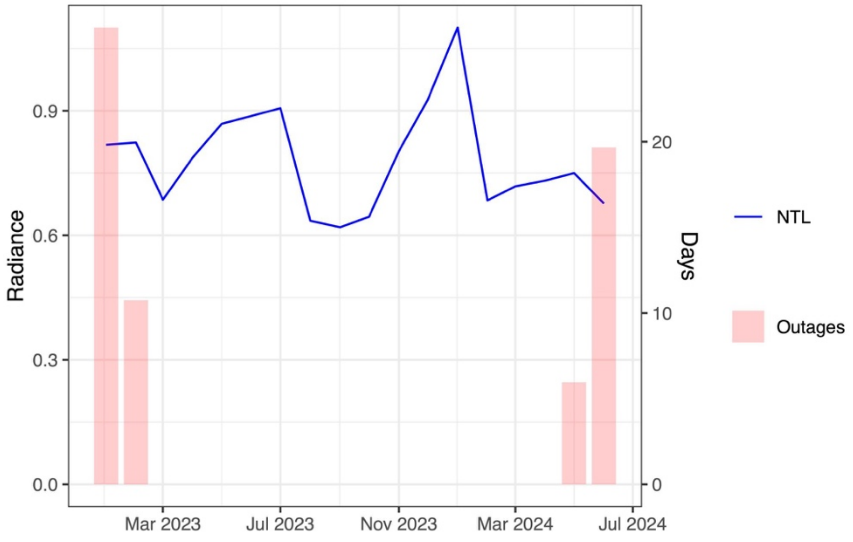

Fig. S21. Mean NTL and days of stabilization blackouts in Ukraine. Blackouts are reported hourly and the total hours were aggregated by month and divided by 24 to determine the total number of days per month. Over this period, blackouts affected most or all regions in the country. Data were not available for 2022. The mean NTL time series is for all of Ukraine.

SI References

1. Wang Z, Shrestha RM, Román MO, Kalb VL. NASA's Black Marble Multiangle Nighttime Lights Temporal Composites. IEEE Geoscience and Remote Sensing Letters. 2022;19:1–5.

2. Iddawela Y. Not all nightlight datasets are the same: Introducing NASA's Black Marble dataset [Internet]. 2023 [cited 2025 Mar 6]. Available from: https://www.spatialedge.co/p/not-all-nightlight-datasets-are-the

3. Marty R, Vicente GS. blackmarbler: Black Marble Data and Statistics [Internet]. 2025 [cited 2025 Mar 24]. Available from: https://cran.r-project.org/web/packages/blackmarbler/index.html

4. Wood S. mgcv: Mixed GAM Computation Vehicle with Automatic Smoothness Estimation [Internet]. 2023 [cited 2025 Jan 7]. Available from: https://cran.r-project.org/web/packages/mgcv/index.html

5. Tarnas MC, Al-Dheeb N, Zaman MH, Parker DM. Association between air raids and reported incidence of cholera in Yemen, 2016–19: an ecological modelling study. The Lancet Global Health. 2023 Dec 1;11(12):e1955–63.

6. IOM. Annual DTM Report on Displacement 2023 [Internet]. International Organization for Migration Displacement Tracking Matrix; 2024 Apr. Available from: https://dtm.iom.int/reports/yemen-annual-dtm-report-displacement-2023
